# Optimization of a wet-cell electrolyzer for efficient oxyhydrogen (HHO) gas production: a step towards sustainable green energy solutions

**DOI:** 10.1038/s41598-026-45418-z

**Published:** 2026-04-15

**Authors:** Nouralddin H. A. Fayez, Mohamed Qenawy, Hassan M. M. Mustafa, Mohamed Shehadeh, Mohamed Taha, A. H. Abdelbaky Elbatran

**Affiliations:** 1 Mechanical Engineering Department, College of Engineering and Technology, Arab Academy for Science, Technology and Maritime Transport, Sadat Road, P.O. Box 11, Aswan, Egypt; 2 Mechanical Engineering Department, Faculty of Energy Engineering, Aswan University, Aswan, 81528 Egypt; 3 Mechanical Engineering Department, Engineering and Renewable Energy Research Institute, National Research Centre, Giza, 12622 Egypt; 4 Faculty of Engineering, Arab Academy for Science, Technology and Maritime Transport, Alexandria, 1029 Egypt; 5 Faculty of Engineering, Arab Academy for Science, Technology and Maritime Transport, Alamein, 1029 Egypt

**Keywords:** HHO Generators, Wet Cell, Fuel, Electrolysis, Green Hydrogen, Energy science and technology, Engineering, Environmental sciences

## Abstract

The urgent need for sustainable energy solutions drives innovation in clean fuel technologies. Oxyhydrogen (HHO) gas, produced through water electrolysis, presents a promising green energy vector. While often studied in dry-cell configurations, wet-cell electrolyzers offer advantages for efficient, scalable production but require further optimization. This study systematically investigates the design and operational parameters of a wet-cell HHO generator to maximize efficiency. Four distinct configurations (Alpha, Beta, Gamma, Delta) were constructed, varying in electrode cross-sectional area (75 × 75 mm² vs. 150 × 150 mm²), plate configuration (18 vs. 20 plates), and tested at different potassium hydroxide (KOH) concentrations (10 and 20 g/L). Performance was evaluated based on HHO flow rate, specific energy consumption, and overall system efficiency. Experimental results demonstrate that the Delta generator significantly outperformed all other designs. It achieved a peak HHO flow rate of 3.4 L/min with a specific energy consumption of 3.1 kWh·m⁻³ and a notable overall system efficiency of 59.74%. In contrast, the Alpha, Beta, and Gamma generators attained lower efficiencies of 12.7%, 23.86%, and 41.9%, respectively. The superior performance of the Delta design is attributed to its optimized combination of a larger electrode area, which reduces current density and associated overpotentials, and an effective electrode configuration that maximizes active surface area and thermal management. This study conclusively identifies optimal electrode geometry and electrolyte management as critical drivers for efficient HHO production. The optimized wet-cell electrolyzer presents a sustainable and practical technology for on-demand green hydrogen production, with direct potential applications as a combustion enhancer or a storage solution for intermittent renewable energy, contributing to the advancement of sustainable energy systems.

## Introduction

Water is the most prevalent substance on Earth and the fundamental source of life. It is also a compound of significant energetic interest, as its molecule consists of hydrogen and oxygen. Hydrogen, the most abundant element in the cosmos, is not found in its pure form on Earth and requires energy for separation. With the escalating costs and environmental drawbacks of conventional fossil fuels, researchers worldwide are intensifying efforts to enhance internal combustion engine efficiency and reduce emissions through sustainable alternatives. Replacing hydrocarbons with renewable, eco-friendly fuels is a major focus. In this context, hydrogen stands out as a promising substitute due to its combustion properties, which yield far fewer hazardous pollutants than hydrocarbon fuels^1,2^. Electrolysis, the process of separating hydrogen from water, currently accounts for 12–15% of global hydrogen production. When optimized to produce a stoichiometric mixture of hydrogen and oxygen, this process generates hydroxy gas, commonly referred to as oxyhydrogen or HHO gas—a colorless, highly combustible, and reactive substance^3^.

The efficiency of electrochemical water electrolysis is fundamentally governed by three key overpotentials: activation, ohmic, and concentration. Activation overpotential arises from the energy barrier for electron transfer during the hydrogen evolution reaction and oxygen evolution reaction and is strongly dependent on the electrocatalyst’s intrinsic activity. Ohmic overpotential is caused by resistance to ion and electron flow within the electrolyte, electrodes, and their interfaces, increasing proportionally with current density. Concentration overpotential results from mass transport limitations that lead to reactant depletion or product accumulation at the electrode surface, an effect that becomes significant at high current densities and is often exacerbated by gas bubble formation^4,5^. The interplay of these overpotentials determines the efficiency and energy consumption of the electrolyzer.

Electrode design, particularly the active area, critically influences these losses. A larger electrode area reduces the current density for a given total current, thereby lowering both activation and concentration overpotentials. For instance, asymmetric electrode configurations with larger anode areas can stabilize the interfacial pH and reduce concentration losses in alkaline electrolysis^6^. Furthermore, increasing the electrochemically active surface area through nano-structuring or the use of catalytic suspensions enhances the number of active sites, effectively reducing activation overpotential and enabling higher current densities at lower applied voltages^7^. Therefore, optimizing water electrolysis performance requires a holistic approach that balances geometric design with material properties to mitigate all three overpotential losses simultaneously.

Substantial research has been dedicated to understanding and improving the electrolysis process for HHO gas production. Studies confirm that systematic investigation into parameters such as catalyst composition, electrolyte selection, and electrode material is crucial for enhancing production rates and overall system efficiency^8^. The development of specialized HHO generator kits has demonstrated significant improvements in gas yield^9^. Catalysts play an essential role in reducing the activation energy of the reaction, while electrolytes like alkaline or acidic solutions improve ionic conductivity, though they may introduce challenges such as material corrosion^10,11^. Physical design parameters, including electrode spacing and alignment, directly impact internal resistance and bubble dynamics, influencing ohmic losses and mass transport^11^. Operational conditions like temperature and pressure also have profound effects; higher temperatures generally improve reaction kinetics and ion mobility^12^ but also affect energy balance, while pressure influences gas bubble behavior and electrolyte resistivity^13^.

Much of this prior work has focused on dry-cell electrolyzer designs. For example, Mahrous et al.^14^ examined the impact of input power, electrolyte concentration, and electrode spacing on gas production and efficiency, noting that efficiency declined beyond a certain input voltage. Olivares et al.^15^ tested various stainless-steel grades (304 L, 316 L, 430) in a dry cell with different KOH and NaOH solutions, identifying 316 L as the most effective cathode material in a KOH electrolyte. Mazloomi et al.^16^ provided a comprehensive analysis of parameters for minimizing energy loss in dry-cell electrolysis, including electrolyte concentration, temperature, electrode spacing, material, and voltage. Ismail et al.^17^ simulated the performance of an HHO dry cell, finding cell efficiency up to 96% with increased temperature and lower supply current, and noting that higher electrolyte concentration increased gas generation. Almassri et al.^18^ evaluated HHO production using ammonium hydroxide (NH₄OH) electrolyte, reporting increased productivity with longer reaction time and higher power, but also significant electrode corrosion.

While dry-cell configurations have been extensively studied, research on wet-cell electrolyzers remains comparatively limited. The wet-cell design offers a distinct advantage: the complete immersion of electrodes in the electrolyte solution provides a larger and more consistent active surface area. This characteristic can lead to higher HHO gas production rates under identical design and operational conditions compared to dry cells^19^. The performance of a wet-cell generator is governed by a complex array of interacting parameters, including electrolyte type and concentration, applied electrical current and voltage, operational temperature and duration, the number and configuration of electrodes (anodes, cathodes, and neutral plates), the cross-sectional area of the plates, and the distance between them. To address this gap, this study aims to systematically optimize the design and performance of a wet-cell HHO generator. The investigation focuses on three critical, interdependent parameters: plate cross-sectional area, electrolyte concentration, and electrode configuration. Through experimental analysis of four distinct generator designs (Alpha, Beta, Gamma, and Delta), the research seeks to identify the optimal configuration that maximizes HHO gas flow rate while minimizing electrical energy consumption per unit of gas produced.

## Methodology

### Electrolyzer design and experiment setup

Electrolysis is an electrochemical process that uses direct current to decompose water (H₂O) into its constituent gases, hydrogen (H₂) and oxygen (O₂), at the cathode and anode, respectively. The volumetric yield of hydrogen is approximately twice that of oxygen, consistent with the stoichiometry of water splitting^3^. This study employs a parallel-plate wet-cell design, where electrodes are fully immersed in an electrolyte. This configuration offers advantages over dry-cell systems, including a larger and more consistent active surface area, improved stability, and simpler construction, which can translate to higher gas production rates^19^.

**Fig. 1:**
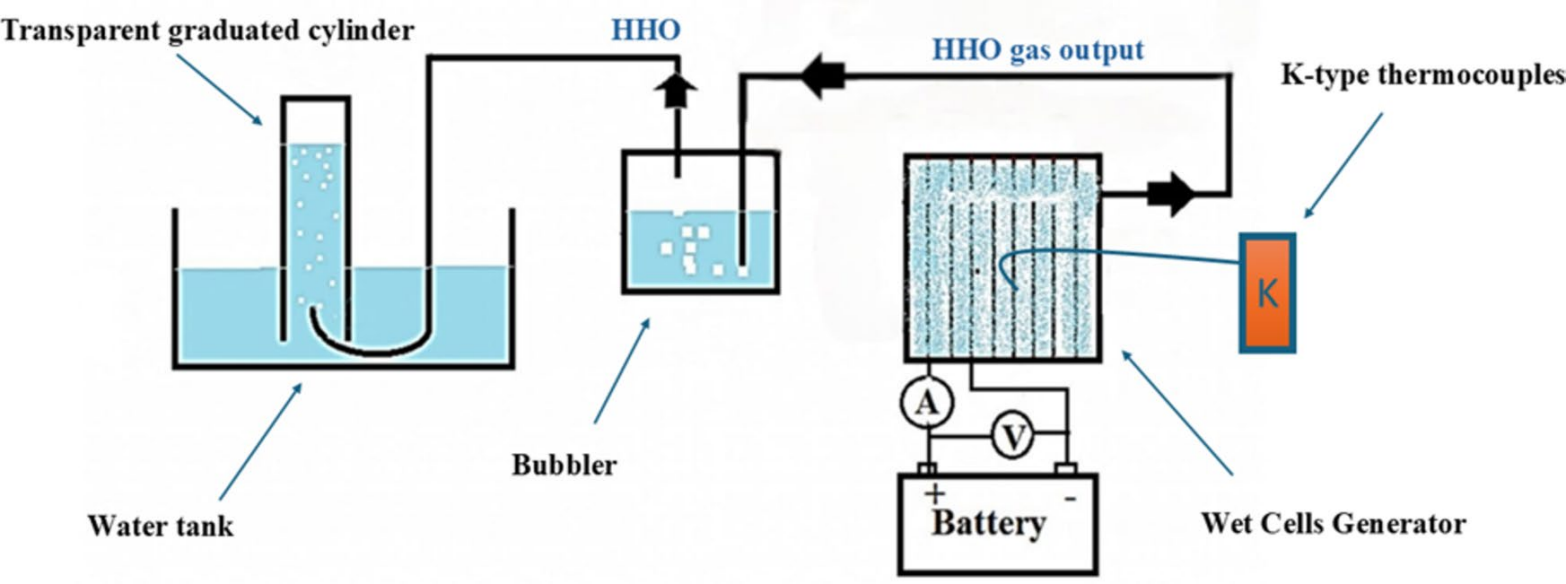
Schematic diagram of the test rig.

The HHO production system, illustrated in Fig. 1, consists of the electrolyzer, a 12 V DC power source (a 60 Ah battery), and a safety and measurement module. The battery provided a constant voltage; consequently, the operating current was determined by the instantaneous internal resistance of the cell, which varied with electrolyte concentration, temperature, and electrode geometry. Power input was monitored using a digital voltmeter and clamp meter. A flashback arrestor and a water-based bubbler were integrated into the gas line for safety; the bubbler also served to filter impurities and act as a preliminary gas reservoir^19,20^.

#### Electrolyzer fabrication and configurations

Four distinct wet-cell electrolyzers, designated Alpha, Beta, Gamma, and Delta, were constructed and tested. The core components were rectangular electrodes fabricated from 1 mm thick stainless steel 316 L, selected for its corrosion resistance and mechanical stability in alkaline environments^15^. The Alpha & Beta generators featured plates with a cross-sectional area of 75 × 75 mm² (0.005625 m²), while the Gamma & Delta generators featured larger plates of 150 × 150 mm² (0.0225 m²).

**Fig. 2:**
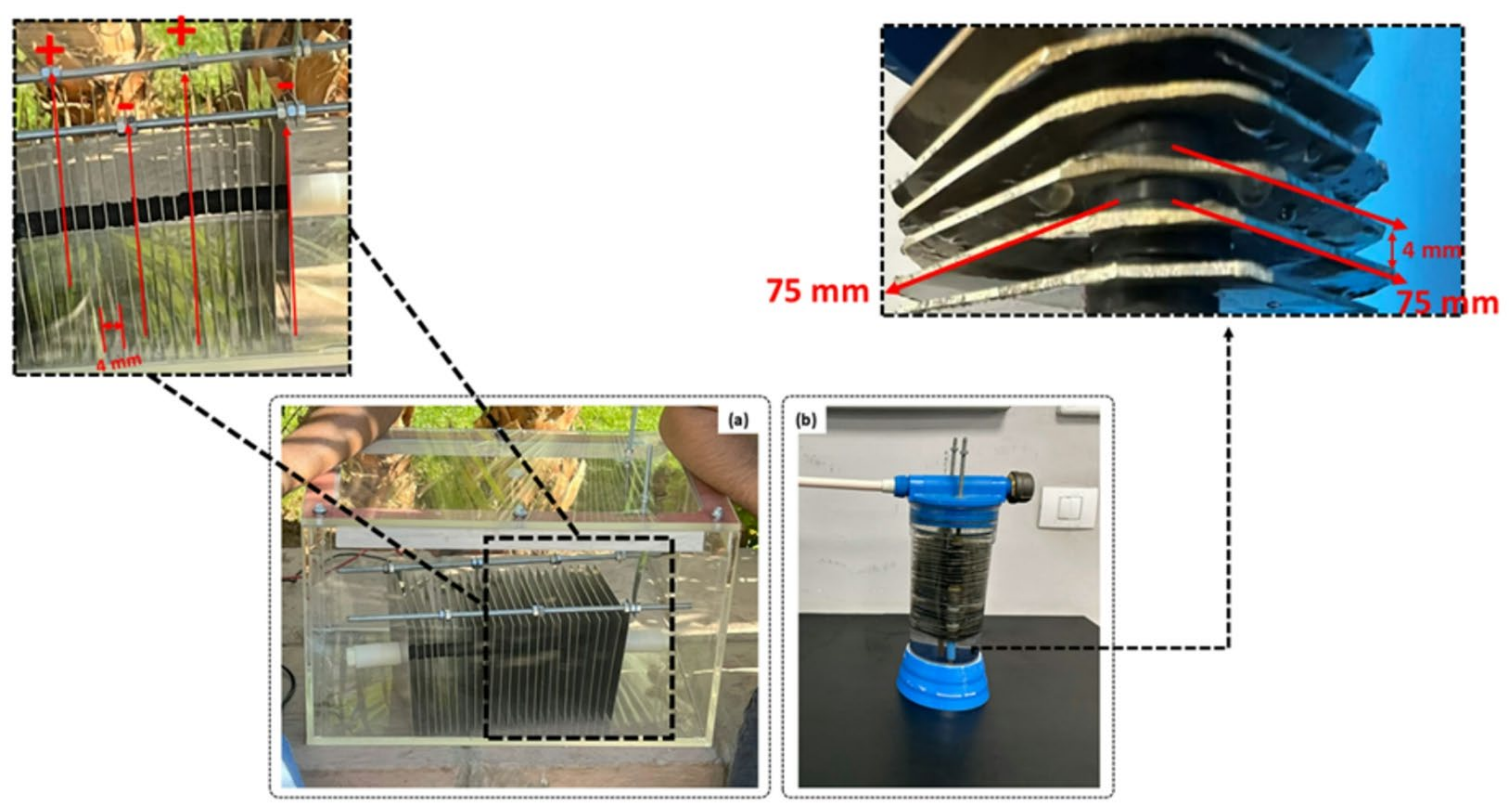
Photograph view of the current wet-cell electrolyzer; (**a**) Gamma & Delta generators and (**b**) Alpha & Beta generators.

The plates were assembled in a parallel stack, separated by 4 mm thick rubber gaskets. These gaskets served as both electrical insulators and seals, defining the inter-electrode gap—a critical parameter influencing ohmic resistance and bubble release. The assembly was secured with threaded rods and sealed within a vessel. The electrode configuration, a key variable in this study, differed among the generators. Alpha & Beta have 18 plates total (2 Anodes, 2 Cathodes, 14 Neutral plates), while Gamma & Delta have 20 plates total (3 Anodes, 3 Cathodes, 14 Neutral plates). In this design, the anodes and cathodes were connected directly to the DC source in parallel. The neutral plates, placed between them, function as bipolar electrodes via induction, effectively increasing the number of electrochemical cells in series within the stack and helping to distribute heat. The physical constructions of the four generators are detailed in Fig. 2, and their key design parameters are summarized in Table 1.

#### Electrolyte preparation

Potassium hydroxide (KOH) was selected as the electrolyte catalyst due to its higher ionic conductivity compared to alternatives like sodium hydroxide (NaOH)^21^. Solutions were prepared at two concentrations: 10 g/L and 20 g/L. Analytical-grade KOH pellets were measured with a digital balance (± 0.01 g) and dissolved in distilled water to the specified volume. Distilled water was used to minimize impurities that could accelerate electrode corrosion^8^. The exothermic dissolution process was conducted with stirring, and the solution was allowed to cool to ambient temperature (25 ± 2 °C) before use. Fresh electrolyte was prepared for each experimental run to ensure consistency.

#### Measurement and data acquisition

The volumetric flow rate of the HHO gas was measured using the water displacement method. Gas from the electrolyzer was fed into an inverted, water-filled 1 L graduated cylinder (± 10 mL resolution). The volume displaced over a measured time interval was recorded. The setup was calibrated, and water levels were equilibrated to minimize hydrostatic error. Flow rate ($\:{\dot{v}}_{HHO}$) was calculated using Eq. (1); where $\:{V}_{HHO}$is the gas volume (L) and $\:t$ is time (min).

1 $$\:{\dot{v}}_{HHO}=\frac{{V}_{HHO}}{t}$$

The applied DC voltage (V) and current (I) were recorded using a digital voltmeter and a clamp meter, respectively. The electrical power input (P) was calculated as per Eq. (2).

2 $$\:P=V\times\:I$$

K-type thermocouple inserted into the electrolyte vessel monitored the operating temperature throughout each experiment.

**Table 1:** Design configuration of HHO wet generators involving KOH catalyst and 4 mm gap distance.

	Number of plates				KOH	Area	Purpose of the study
	Neutral	Cathodes	Anodes	Total			
Alpha	14	2	2	18	10 g/L	75 × 75 mm^2^	• Effect of electrolyte concentration • Effect of electrolyte concentration, the cross-sectional area of the plate, and number of positive and negative electrodes
Beta					20 g/L		
Gamma	14	3	3	20	10 g/L	150 × 150 mm^2^	
Delta					20 g/L		

### Experimental procedure and performance evaluation

The study focused on evaluating the effect of three controlled parameters: electrode configuration (four designs), electrolyte concentration (10 and 20 g/L KOH), and plate cross-sectional area (two sizes). The response variables were HHO flow rate, power consumption, specific energy, and system efficiency.

Each generator configuration was tested at both electrolyte concentrations. For a given test, the system was operated continuously for one hour under constant voltage (12 V) to reach a steady operational state. Voltage, current, electrolyte temperature, and produced gas volume were recorded at 5-minute intervals. Each experimental condition was repeated three times, and the reported results are the arithmetic mean of these replicates. This approach mitigated the influence of transient fluctuations.

For the specific energy consumption ($\:{E}_{s\:}$), the electrical energy required to produce a unit volume of HHO gas, indicating the process’s practical efficiency. The flow rate was first converted to m³/h through Eq. (3), then $\:{E}_{s\:}$ is estimated through Eq. (4); where P is power in kW and $\:{\dot{v}}_{HHO}$ is in m³/h.

3 $$\:{\dot{v}}_{HHO}\left(\frac{{\mathrm{m}}^{3}}{\mathrm{h}}\right)={\dot{v}}_{HHO}\left(\mathrm{L/min}\right)\times\:60\times\:{10}^{-3}$$

4 $$\:{E}_{s\:}=\frac{P}{{\dot{v}}_{HHO}}\:$$

The overall system efficiency ($\:{\upeta\:}$), which evaluates the total energy conversion effectiveness of the practical HHO generator system^22^, is accounting for all electrical and thermal losses. It is defined in Eq. (5) as the ratio of the chemical energy content of the produced hydrogen (which constitutes 2/3 of the HHO volume^23^ to the electrical energy input^24^.

5 $$\:{\upeta\:}=\frac{{\dot{v}}_{{H}_{2}}\:\times\:{\rho\:}_{{H}_{2}}\times\:{LHV}_{{H}_{2}}}{P}\:\times\:100$$

Here; $\:{\dot{v}}_{{H}_{2}}$ = 0.66 × $\:{V}_{HHO}$ is the hydrogen flow rate (m³/s), $\:{\rho\:}_{{H}_{2}}$ = 0.0838 kg/m³ is the hydrogen density, $\:{LHV}_{{H}_{2}}$= 120 × 10⁶ J/kg is the lower heating value of hydrogen, and P is the electrical power input in Watts (W). It is important to note that Eq. (5) assumes the generated HHO gas maintains the ideal stoichiometric composition of 2:1 hydrogen to oxygen by volume (≈ 66% H₂). In practice, slight deviations from this ratio may occur due to factors such as gas dissolution, recombination reactions, or measurement uncertainties in real-time gas collection. Such deviations would affect the calculated heating value and, consequently, the accuracy of the reported efficiency.

### Uncertainty analysis

Measurement uncertainties, arising from instrument accuracy, calibration, and environmental factors, were systematically evaluated using the method described by Heywood^25^. The total uncertainty $\:{U}_{R}$ for a calculated parameter $\:R$ (e.g., efficiency, specific energy), which is a function of independent measured variables $\:{x}_{1}$, $\:{x}_{2}$, $\:{x}_{\mathrm{n}}$was estimated via root-sum-square propagation, as described by Eq. (6).

6 $$\:{U}_{R}=\sqrt{{\left(\frac{\partial\:R}{\partial\:{x}_{1}}{U}_{1}\right)}^{2}+{\left(\frac{\partial\:R}{\partial\:{x}_{2}}{U}_{2}\right)}^{2}+\dots\:+{\left(\frac{\partial\:R}{\partial\:{x}_{n}}{U}_{n}\right)}^{2}}$$

The accuracies and operational ranges of the primary instruments are listed in Table 2. The resulting relative uncertainties for the key derived parameters (HHO flow rate, power, specific energy, and system efficiency) are presented in Table 3. This analysis ensures the reported data are interpreted within statistically valid confidence intervals.

**Table 2:** Accuracy and range of measurement devices.

DC Current	Range	Accuracy
	0.0–400.0 A	± 0.1%
DC Voltage	0.0–600.0 V	-
Stopwatch	-	± 0.2 S
K-type thermocouples	0.0–100 ^◦^C	± 0.4%

**Table 3:** Relative uncertainty of the measured variables.

Power	± 2.5%
Electrolyte Temperature	± 1.77%
Electrolyte Concentration	± 1.56%
HHO gas volume flow rate.	± 4.05%
Generator Efficiency	± 3.88%
The Specific Energy	± 3.50%

## Results and discussions

This section presents the experimental findings from testing four wet-cell HHO generator designs (Alpha, Beta, Gamma, Delta) under varying operational parameters. The analysis systematically examines the influence of electrode configuration, plate cross-sectional area, electrolyte concentration, current, and temperature on key performance metrics: HHO flow rate, specific energy consumption, and overall system efficiency. The discussion integrates these results with fundamental electrochemical principles and benchmarks them against the existing literature.

### Influence of electrode configuration and cross-sectional area on HHO flow rate

**Fig. 3:**
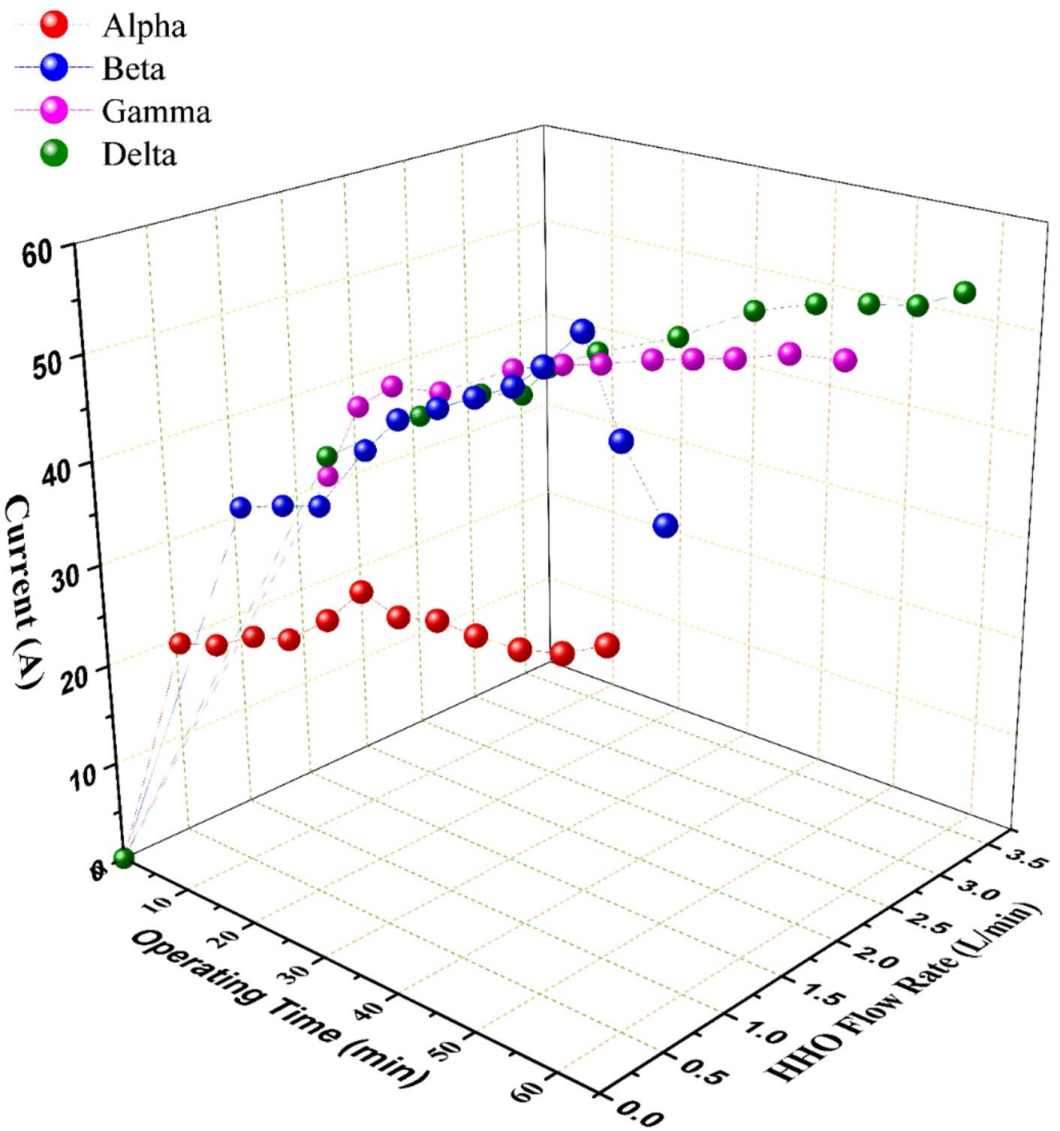
The performance analysis of the wet HHO generators over the operating time with regard the HHO flow rate and the consumed current; data points represent the mean of three experimental runs.

The performance of the four generator configurations, detailed in Table 1, revealed significant disparities in HHO production. As illustrated in Fig. 3, a clear hierarchy of volumetric flow rate was established: Delta > Gamma > Beta > Alpha. This performance gradient is fundamentally linked to two core geometric design parameters: the electrode configuration (i.e., the number of anodes and cathodes) and the plate cross-sectional area.

The Delta & Gamma generators, utilizing a [3 Cathodes, 3 Anodes, 14 Neutral plates] configuration, inherently possess a greater number of active electrochemical cells within the stack compared to the [2C2A14N] setup of Alpha & Beta. This increases the total available reaction surface area. Furthermore, Delta & Gamma employ plates with a substantially larger cross-sectional area (150 × 150 mm²) versus the 75 × 75 mm² plates of Alpha & Beta.

The synergistic effect of these design choices is a critical reduction in the operational current density (current per unit electrode area). Electrochemical theory dictates that lower current density minimizes the activation overpotential required to drive the hydrogen and oxygen evolution reactions and reduces concentration polarization caused by mass transport limitations of reactants and products^26,27^. Consequently, for a given total current, the larger-area configurations (Gamma, Delta) operate in a more thermodynamically favorable regime, translating directly to enhanced gas production rates for comparable electrical input, as unequivocally demonstrated in Fig. 3.

The vertical orientation of the electrode plates, particularly beneficial for the larger plates in Gamma & Delta (Fig. 2a), promotes buoyancy-driven detachment of gas bubbles. Efficient bubble release is paramount to mitigating the “bubble effect,” wherein adherent gas bubbles insulate the electrode surface, increase ohmic resistance, and diminish the effective electrochemically active surface area^28^. The Delta generator’s design, therefore, achieves an optimal balance: it maximizes active surface area, minimizes detrimental current density, and ensures efficient evacuation of reaction products^29^.

### Effect of electrolyte concentration on HHO gas production

The ionic conductivity of the electrolyte, governed by its type and concentration, is a primary determinant of electrolyzer performance. Potassium hydroxide (KOH) was selected for its superior ionic conductivity compared to alternatives like sodium hydroxide (NaOH)^21^. Experimental results, presented in Figs. 3 and 5, confirm that increasing the KOH concentration from 10 g/L to 20 g/L consistently enhanced the HHO flow rate across all four generator designs.

**Fig. 4:**
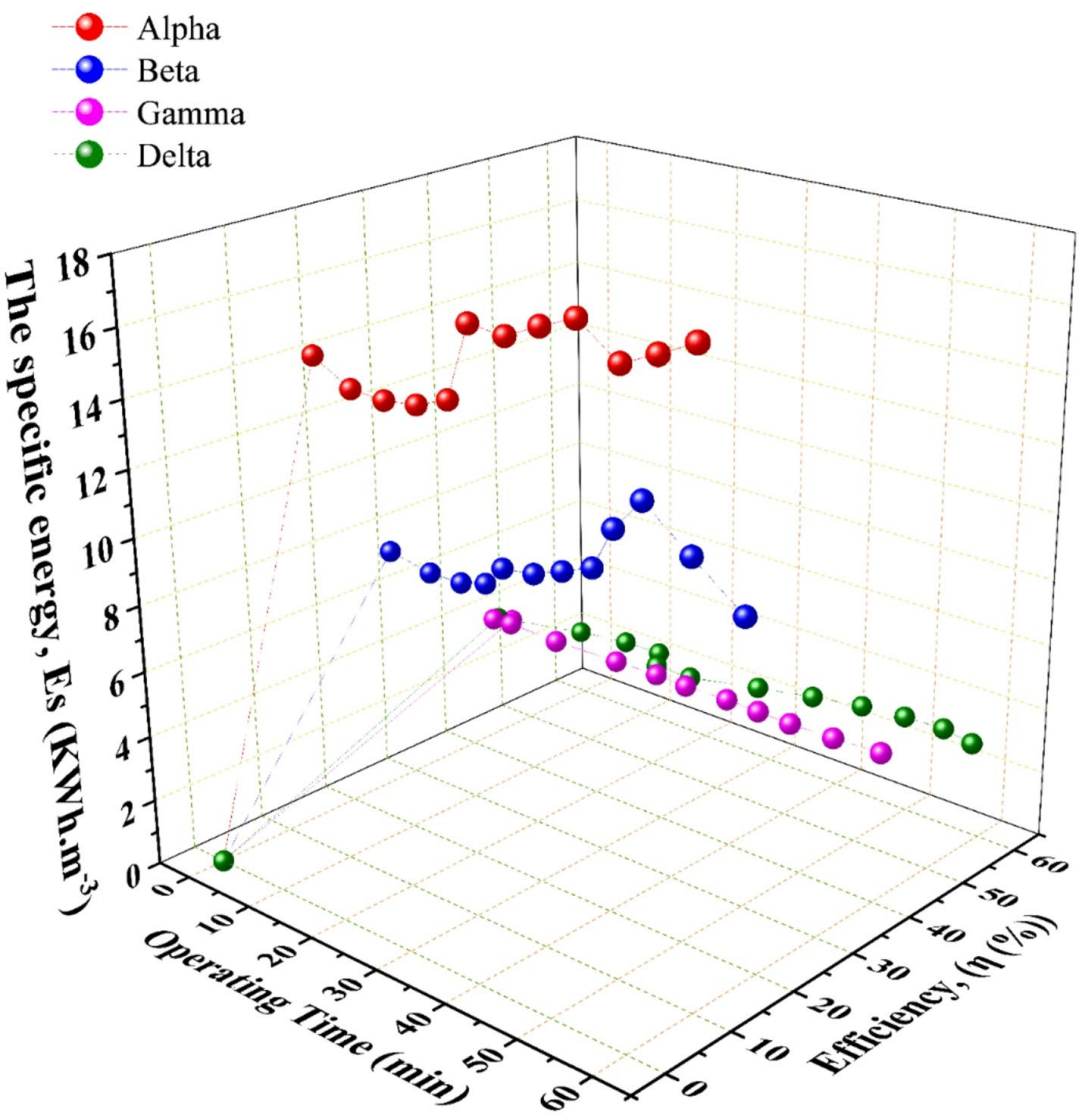
The performance analysis of the wet HHO generators over the operating time with regard to the generator’s overall efficiency and the specific energy; data points represent the mean of three experimental runs.

**Fig. 5:**
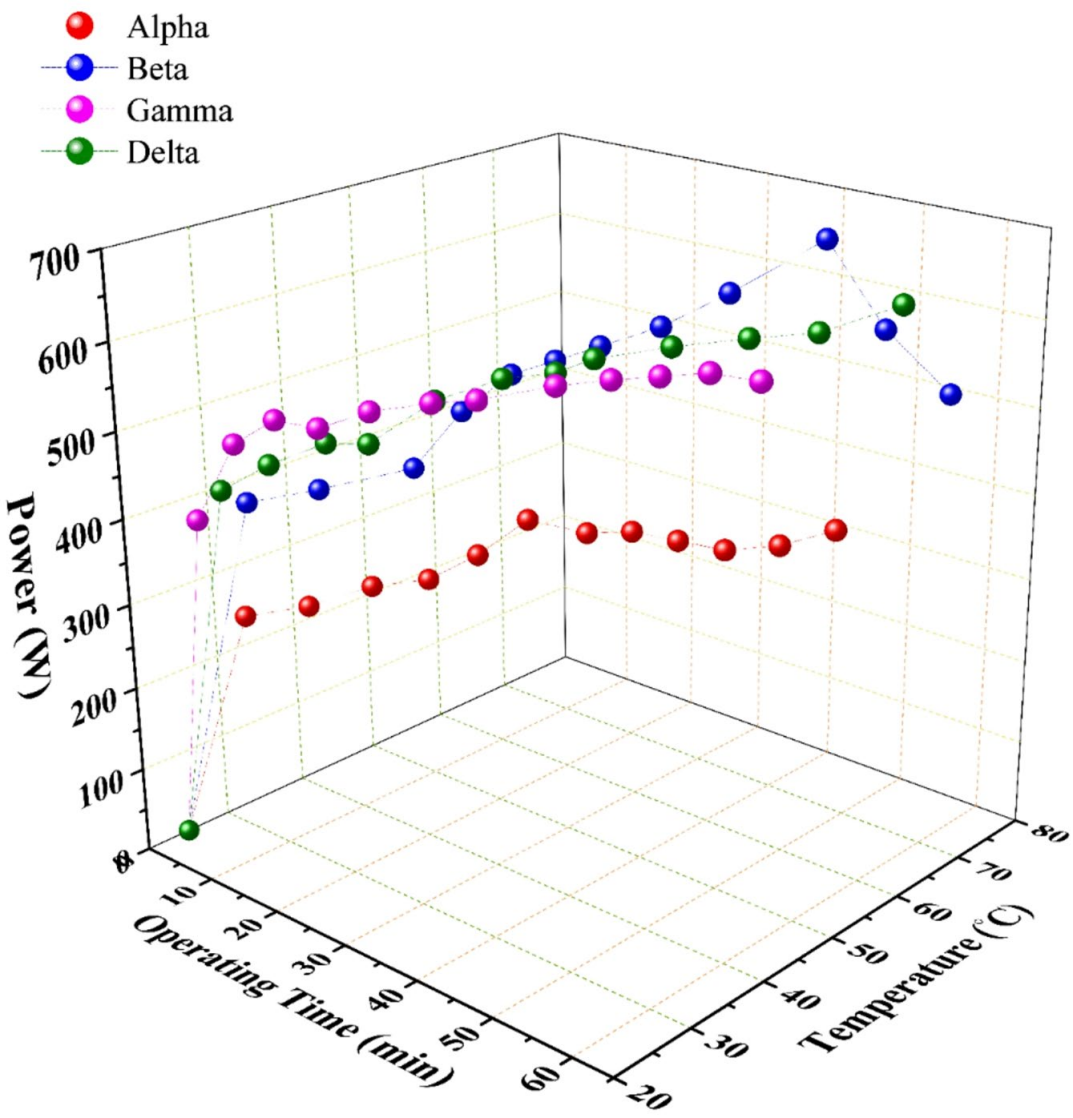
The performance analysis of the wet HHO generators over the operating time with regard the production temperature and the input power; data points represent the mean of three experimental runs.

This enhancement is attributed to a higher concentration of mobile charge carriers (K⁺ and OH⁻ ions), which lowers the ohmic resistance of the electrolyte solution. A reduced ohmic drop means a larger fraction of the applied terminal voltage is available to drive the Faradaic reactions at the electrode-electrolyte interface. This leads to an increased current draw at a constant applied voltage (12 V DC), and by Faraday’s first law of electrolysis, a directly proportional increase in gas production rate. It is critical to note, however, that this increased production comes with elevated current and total power consumption, as evidenced in Fig. 5. The Delta generator, with its robust design and superior heat dissipation, leveraged the higher-concentration electrolyte most effectively, achieving the highest absolute flow rates.

### Interplay of consumed current, temperature, and HHO flow rate

Under the constant-voltage power supply, the current drawn by each generator was an emergent property of its instantaneous internal resistance, itself a function of design, electrolyte concentration, and notably, operating temperature.

**Fig. 6:**
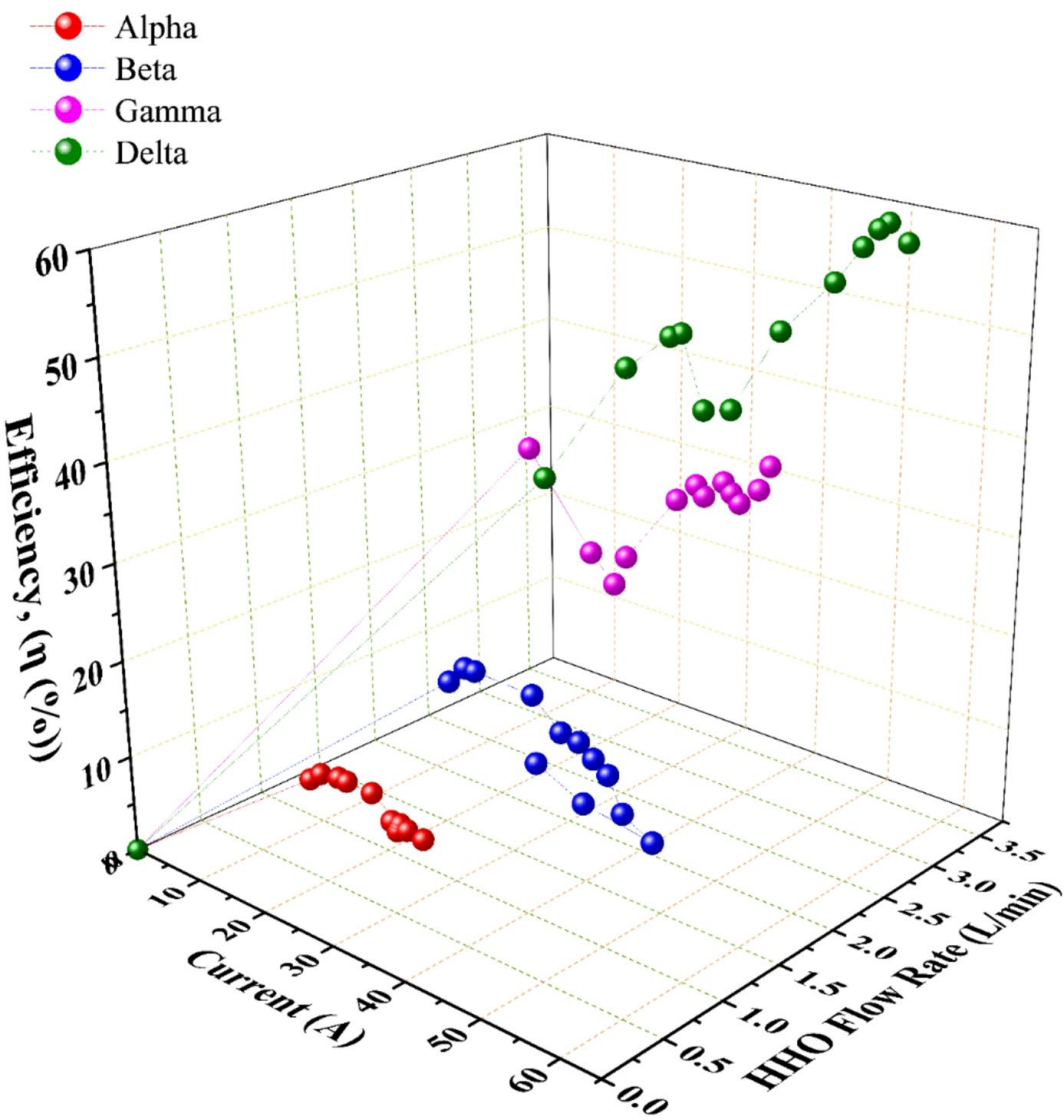
The change of the consumed current with overall efficiency and generated HHO flow rate for different generators; data points represent the mean of three experimental runs.

For current and flow rate, Fig. 6 demonstrates a strong positive correlation between the consumed current and the HHO flow rate for all configurations, in accordance with Faraday’s law. Delta generator sustained the highest average current draw (~ 43 A) and consequently produced the highest rate. However, a critical divergence emerges when examining efficiency metrics between small-area (Alpha, Beta) and large-area (Gamma, Delta) generators.

For thermodynamics and efficiency trade-offs, the electrolysis process is inherently exothermic; a portion of the input electrical energy is invariably dissipated as heat. As shown in Fig. 5, operational temperature increased steadily during testing for all generators. This temperature rise has dual, competing consequences: it beneficially increases ionic mobility and accelerates reaction kinetics (as described by the Arrhenius equation)^30^, but it can detrimentally lead to excessive water evaporation, changes in electrolyte concentration, and increased parasitic current pathways if not properly managed^31^.

**Fig. 7:**
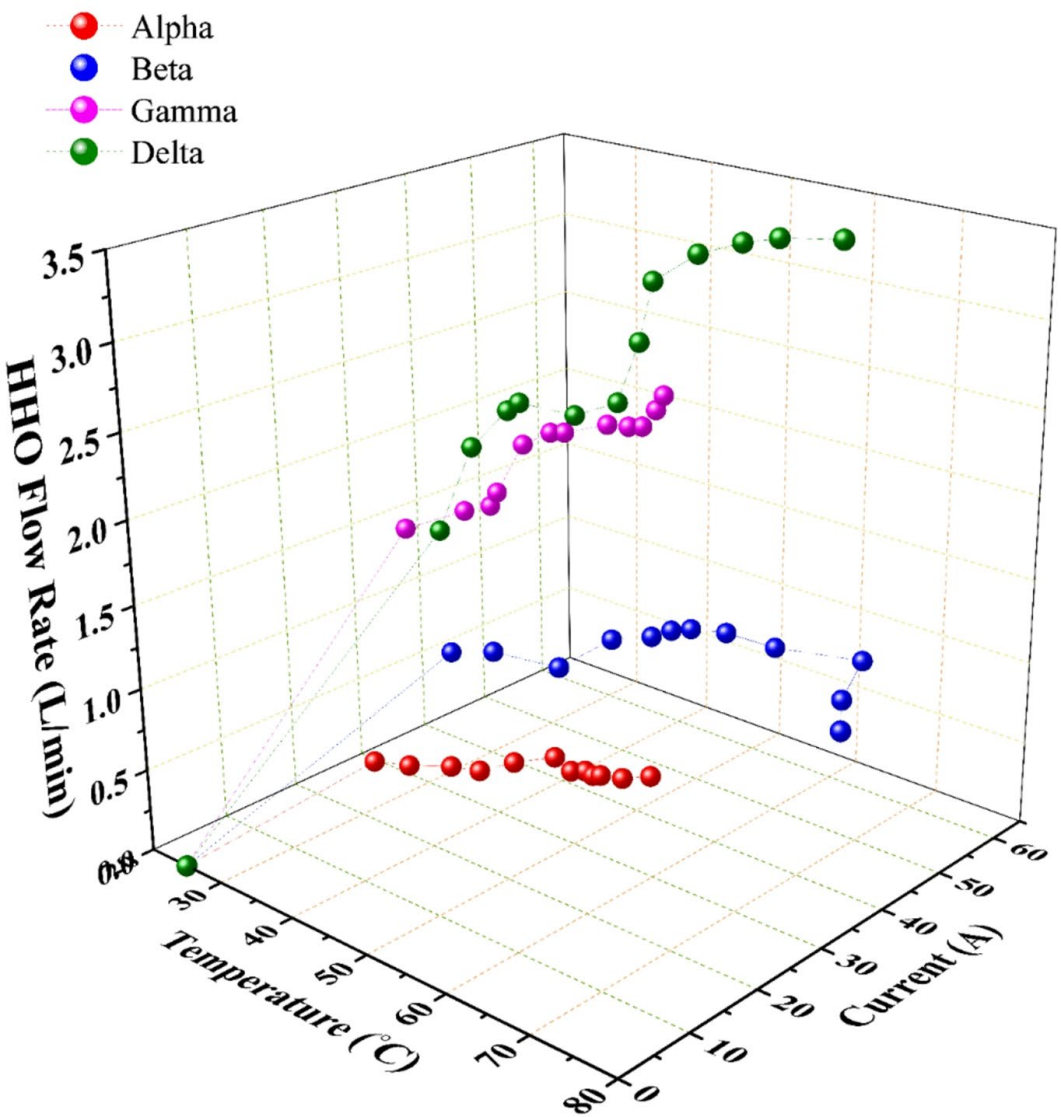
The change of the production temperature with the generated HHO flow rate and the consumed current; data points represent the mean of three experimental runs.

The Beta generator exemplified a worst-case thermal scenario. Its combination of smaller plate area and [2C2A14N] configuration led to poor heat dissipation, causing the electrolyte temperature to escalate to 72 °C (Fig. 5) (Fig. 7). This indicates a state of significant energy waste, where a large fraction of the input current (≈ 44 A) was converted into Joule heating rather than productive electrolysis, severely undermining its operational efficiency.

For the specific energy consumption, this metric, where the electrical energy required to produce a unit volume of HHO gas, is a paramount indicator of practical efficiency. The trends in $\:Es$, depicted in Figs. 4 and 9, reveal a pivotal finding. For the large-area Gamma & Delta generators, increasing current (and thus flow rate) led to a progressive decrease in specific energy consumption. This signifies that gas production scaled more efficiently than power input, a hallmark of a well-designed system operating within its optimal range. For the small-area Alpha & Beta generators, increased current often resulted in a rise in $\:Es$. This indicates diminishing returns and inefficiency, where additional electrical power is disproportionately lost as heat, consistent with the observed thermal runaway in the Beta generator. This phenomenon aligns with findings reported by Wu et al.^32^ and Hassan et al.^31^, who attributed similar efficiency drops to electrolyte heating.

**Fig. 8:**
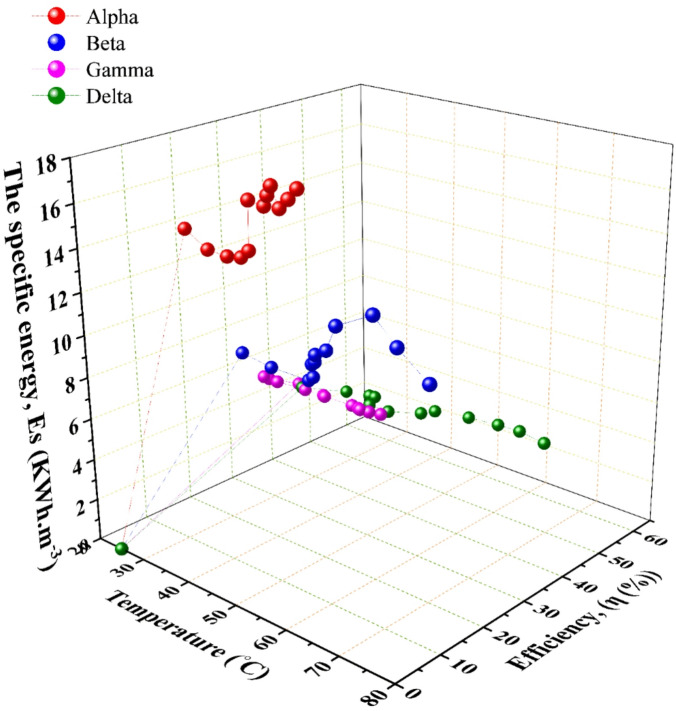
The change of the Production Temperature with the generator’s overall efficiency and the specific energy; data points represent the mean of three experimental runs.

### Overall system efficiency and identification of the optimal configuration

The overall system efficiency, calculated as the ratio of the chemical energy content of the produced hydrogen to the total electrical energy input, provides the most holistic performance assessment. The results, summarized in Fig. 8; Table 4, are conclusive. The Delta generator achieved the highest recorded peak efficiency of 59.74%, concurrently attaining a specific energy consumption of 3.1 kWh·m⁻³. This superior performance is the direct result of its optimized synthesis of design parameters. A Large cross-sectional area minimizes current density overpotentials, while the [3C3A14N] configuration maximizes the total active electrochemical surface area. Substantial electrolyte volume (45 L) provides significant thermal mass, enabling effective heat management and preventing the efficiency-degrading thermal runaway observed in the Beta generator. In contrast, the Alpha & Beta generators demonstrated lower and less stable efficiencies, with Beta’s performance notably declining as operational temperature exceeded its optimal range. The Gamma generator performed robustly but was consistently outperformed by Delta, underscoring the incremental benefit of Delta’s specific geometric and configurational advantages.

**Fig. 9:**
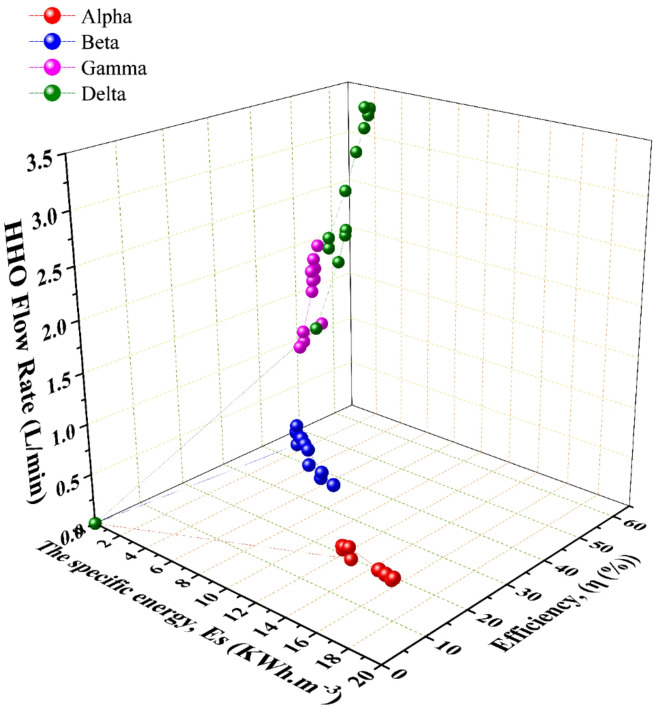
The change of the specific energy with the generators overall efficiency and generated HHO flow rate; data points represent the mean of three experimental runs.

### Safety considerations and gas quality for practical application

The practical deployment of HHO generation systems, especially in mobile contexts, necessitates rigorous safety protocols. Oxyhydrogen gas possesses a wide flammability range (4–94% vol in air) and high flame speed, mandating engineered safeguards^33,34^. In this study, primary safety was ensured through a water-based bubbler, which acted as both a flashback arrestor and a filter for electrolyte mist^35,36^.

The gas produced is a stoichiometric mixture of H₂ and O₂, saturated with water vapor at the operating temperature. While suitable for direct combustion in many applications, integration with sensitive engine control systems or storage would require auxiliary drying and purification stages to prevent condensation and corrosion^37,38^. Crucially, for any real-world application, an integrated safety philosophy is required—incorporating flame arrestors, pressure/temperature sensors, and fail-safe electrical controls—to ensure on-demand generation without the hazards associated with HHO storage^35,36^.

### Scalability, manufacturing, and integration challenges

Transitioning the optimized Delta design from a laboratory prototype to a scalable technology involves several considerations. For materials and cost, the prototype utilizes commercially available materials (316 L SS, acrylic, EPDM). A preliminary bill of materials (Table 5) estimates a cost of ≈ 207 USD, suggesting a pathway to cost-effective manufacturing at scale. For manufacturing, scaling to higher capacities requires addressing challenges in maintaining uniform compression and sealing across larger plate stacks. A modular approach, employing multiple standardized Delta-type units in parallel, could mitigate these issues. For renewable integration, it could be used with intermittent sources like solar PV, the integration of a power conditioning unit is essential to adapt the variable power supply to the electrolyzer’s optimal operating point, ensuring sustained high efficiency.

### Environmental impact and carbon footprint considerations

The environmental merit of electrolytic hydrogen production is intrinsically tied to the carbon intensity of the electricity source. The high system efficiency (59.74%) of the Delta generator directly reduces the amount of electricity required per unit of hydrogen produced. When this electricity is sourced from renewables (e.g., solar, wind), the system’s operational carbon footprint can be minimized, aligning with Sustainable Development Goals for clean energy^39–42^. While a full life-cycle assessment is beyond this study’s scope, the use of common materials like stainless steel suggests a manageable embodied energy footprint compared to systems relying on scarce catalyst materials.

### Comparative analysis and technological competitiveness

A normalized performance comparison, presented in Table 4, facilitates an equitable assessment across the different generator scales. Metrics such as volumetric yield per ampere and areal production rate confirm the intrinsic efficiency of the Delta design.

Table 6 provides a benchmark against prior HHO generator studies. The Delta generator’s performance—with an efficiency nearing 60% and a specific energy of 3.1 kWh·m⁻³—places it at the forefront of reported wet-cell and dry-cell systems in the literature. Many existing designs, particularly dry-cell variants, are hampered by inferior heat dissipation and bubble management, leading to lower sustained efficiencies.

Furthermore, when considering system-level efficiency—the metric most relevant for applied engineering—the Delta generator’s ~ 60% efficiency is competitive with the lower to middle range of commercial alkaline and PEM electrolyzer systems (typically 45–65% system efficiency) as shown in Table 7,^43–46^. It is crucial to note that commercial systems often produce pure, pressurized H₂ for storage and distribution, whereas the Delta generator produces mixed HHO gas at near-ambient pressure for immediate use, representing a different, often more direct, application pathway.

**Table 4:** Comparison of different HHO wet cells performance and productivity; values represent the mean ± standard deviation calculated from three independent experimental runs.

Efficiency (%)	Alpha	Beta	Gamma	Delta
	13.27 ± 0.52	23.86 ± 0.93	41.9 ± 1.63	59.74 ± 2.32
Specific energy (KWh.m^− 3^)	13.89 ± 0.49	7.73 ± 0.27	4.4 ± 0.15	3.1 ± 0.11
Flow rate (L/min [m³/h])	0.36 [0.0216] ± 0.015	0.88 [0.0528] ± 0.036	1.5 [0.09] ± 0.061	3.37 [0.2022] ± 0.136
Yield per current (L·min⁻¹·A⁻¹)	0.0144	0.0259	0.0455	0.0648
Areal production rate (L·min⁻¹·cm⁻²)	1.88 × 10⁻⁴	4.60 × 10⁻⁴	1.75 × 10⁻⁴	3.94 × 10⁻⁴
Current (A)	25	34	33	52
Power (W)	300	408	396	624
Temperature (^◦^C)	38	36	25	58

**Table 5:** Approximate cost of Delta HHO production.

generator	
HHO generator kit parts	**Approximate cost ($)**
Acrylic tank	50
Stainless steel 316 L plates	75
Rubber gaskets (4 mm)	5
Stainless steel bolts and screws	2
Bubbler	3
Connecting wires	10
K-type thermocouples	7
digital clamp meter	55
Total cost	207 $

**Table 6:** Comparison of the enhancement of the current study with the literature.

	Cell	Efficiency	Specific energy	Flow rate	Current
		(%)	(KWh.m^− 3^)	(L/min)	(A)
Najafi et al.^24^	Dry	39.12	4.71	0.3	7
Rusdianasari et al.^47^	-	6.31	29.18	0.1028	15
Kady et al.^48^	Dry	45.25	4.1	0.767	30
Kady et al.^48^	Dry	42.69	4.32	0.82	34
Soly et al.^19^	Wet	40.59	4.54	1.376	60
Soly et al.^19^	Dry	36.87	5	1.25	60
Mousa et al.^49^	Dry	53.79	3.43	0.3439	5.9
Current study (Delta)	Wet	59.74	3.1	3.37	52

**Table 7:** Positioning Delta vs. HHO and commercial electrolyzers.

Technology/System	Output	Efficiency (%)	Specific energy (kWh/Nm³)	Typical Scale
Current study (Delta)	HHO (H₂+O₂)	~ 60	~ 3.1 kWh/m³ HHO	Lab/small device
HHO lab generator^46^	HHO (H₂+O₂)	53.8–59	3.43–3.5 kWh/m³ HHO	Lab
HHO commercial^50^	HHO (H₂+O₂)	21–40	4–6 kWh/m³ HHO	Retrofit/vehicle
Alkaline electrolyzer ^44-45^	H₂ (pure)	45–65	4.0–4.5 kWh/Nm³ H₂	Industrial (MW)
PEM electrolyzer ^44-45^	H₂ (pure)	50–65	4.5–5.0 kWh/Nm³ H₂	Industrial (MW)
AEM electrolyzer ^44, 51-53^	H₂ (pure)	45–60	4.5–5.5 kWh/Nm³ H₂	Pilot/industrial

## Conclusion

This study successfully optimized the design and operation of a parallel-plate wet-cell electrolyzer for efficient oxyhydrogen (HHO) gas production. Four distinct generator configurations (Alpha, Beta, Gamma, Delta) were constructed and evaluated based on electrode cross-sectional area, plate configuration, and electrolyte concentration to determine the optimal balance between high gas yield and low specific energy consumption. The experimental results led to several definitive conclusions:

- The Delta generator emerged as the unequivocally superior design, achieving a peak overall system efficiency of 59.74% and a specific energy consumption of 3.1 kWh per cubic meter of HHO gas. This performance significantly outperformed the Alpha (13.27%, 13.89 kWh·m⁻³), Beta (23.86%, 7.73 kWh·m⁻³), and Gamma (41.9%, 4.4 kWh·m⁻³) configurations.
- Delta’s success is attributed to its synergistic combination of a large electrode cross-sectional area (150 × 150 mm²), which reduces current density and associated overpotentials; an optimized [3 Cathodes, 3 Anodes, 14 Neutral plates] configuration, maximizing the active electrochemical surface area; and effective thermal management enabled by a substantial electrolyte volume, preventing efficiency losses due to overheating.
- The study systematically confirmed that increasing the KOH electrolyte concentration from 10 to 20 g/L enhanced ionic conductivity and raised HHO flow rates for all designs, with Delta utilizing this most effectively. While higher current increased gas production universally (per Faraday’s law), its impact on efficiency was design-dependent. Only the large-area designs (Gamma, Delta) maintained or improved efficiency with higher current, whereas the small-area Beta generator suffered from thermal inefficiency, converting excess power to waste heat. Stable, moderate operating temperature (40–65 °C) is beneficial, but uncontrolled heating (> 70 °C, as in Beta) is detrimental to sustained efficiency.

In summary, this work demonstrates that efficient HHO production in a wet-cell electrolyzer is not governed by a single parameter but by the holistic integration of geometric design, electrode configuration, and operational control. The Delta generator provides a validated blueprint for such an integrated approach, achieving a compelling combination of high gas flow rate (2.42 L/min average) and low energy demand. However, to transition this optimized laboratory prototype into a viable technology for sustainable energy systems, future research should address the following multi-faceted challenges:

- Conduct extended operational studies (> 500 h) to assess performance degradation, electrode corrosion (pitting, fouling), and electrolyte stability under continuous and cyclic loading. This is essential for determining maintenance intervals and lifespan, particularly for automotive or industrial applications.
- Investigate cost-effective, high-activity electrode coatings (e.g., nanostructured Ni-Fe, Ni-Mo, or Co-P alloys) to further reduce the activation overpotential. Research should also explore advanced separator materials and corrosion-resistant alloys to enhance longevity without significantly increasing cost.
- Develop and test integrated systems coupled directly with renewable energy sources (solar PV, wind). This requires engineering robust power electronics and intelligent energy management systems capable of stabilizing electrolysis operation under highly variable input power, maximizing both energy yield and device lifetime.
- Move beyond generic efficiency metrics to tailor the design for specific end-uses. For automotive applications, this means optimizing for engine bay environments (vibration, temperature swings) and integrating comprehensive, failsafe gas handling and control systems. For stationary storage, scaling strategies and hybridization with existing infrastructure should be explored.
- Perform a detailed cost analysis at pilot-scale production and a full cradle-to-grave LCA. This will quantify the true economic viability and environmental footprint (including embodied carbon of materials) of the system when deployed at scale, providing critical data for commercialization and policy support.

By advancing along these research pathways, the optimized wet-cell electrolyzer presented here demonstrates potential to evolve from a laboratory prototype into a component suitable for further evaluation within decentralized hydrogen production contexts. However, extensive validation—including durability testing, safety certification, and integration studies—remains necessary before such applications can be realized.

## Data Availability

Data will be made available on request to the corresponding author (m_qenawy@aswu.edu.eg).
